# Formula constant optimisation techniques including variation of keratometer or corneal refractive index and consideration for classical and modern IOL formulae

**DOI:** 10.1371/journal.pone.0282213

**Published:** 2023-02-24

**Authors:** Achim Langenbucher, Nóra Szentmáry, Alan Cayless, Jascha Wendelstein, Peter Hoffmann

**Affiliations:** 1 Department of Experimental Ophthalmology, Saarland University, Homburg, Saar, Germany; 2 Dr. Rolf M. Schwiete Center for Limbal Stem Cell and Aniridia Research, Saarland University, Homburg, Saar, Germany; 3 Department of Ophthalmology, Semmelweis-University, Budapest, Hungary; 4 School of Physical Sciences, The Open University, Milton Keynes, United Kingdom; 5 Department of Ophthalmology, Johannes Kepler University Linz, Linz, Austria; 6 Augen- und Laserklinik Castrop-Rauxel, Castrop-Rauxel, Germany; University of Warmia, POLAND

## Abstract

**Background:**

To investigate whether variation of the keratometer/corneal refractive index nK/nC improves the performance (prediction error PE) of classical and a modern intraocular lens (IOL) power calculation formula and further, to establish whether any trend error of PE for corneal radius R could be eliminated using formula constant and nK/nC optimisation.

**Methods:**

Based on 2 large datasets (1: N = 888 Hoya Vivinex aberration-correcting and 2: N = 822 Alcon SA60AT spherical lens) a classical formula constant optimisation has been performed for the Hoffer Q, Holladay 1, Haigis and Castrop formulae, to minimise the root mean squared (rms) PE (situation A). In two further optimisations, the formula constants and the formula specific nK/nC value were optimised to minimise the rms PE (situation B) or rms PE and trend error of PE for R (situation C). Nonlinear iterative optimisation strategy was applied according to Levenberg-Marquardt.

**Results:**

Optimising for rms PE and trend error (C) mainly improved the performance of the Holladay 1. The Haigis formula also showed a slight improvement compared to (A). The Hoffer Q formula shows no relevant trend error of PE for R. In contrast, the Holladay shows a positive and the Haigis (and the Castrop a slight) negative trend error of PE for R. The trend error could be fully eliminated by optimising formula constants and nK/nC in (B), but this was at the cost of overall performance in the case of the Holladay 1 formula.

**Conclusion:**

Classical IOL calculation concepts should be critically examined for potential improvement of formula performance by variation of the empirical nK/nC value defined in the formula. With additional degrees of freedom additional optimisation terms such as trend errors might be considered in new intelligent optimisation strategies.

## Introduction

There are 3 basic principles for calculating intraocular lenses in cataract surgery: empirical strategies including linear or nonlinear regressions or machine learning applications, theoretical-optical formulae based on linear Gaussian optics, and numerical raytracing [1–3].

Empirical strategies are independent of an optical model for the pseudophakic eye. In these strategies, the interactions between potential predictors derived from biometry are ‘trained’ on a large dataset of previous cataract surgeries where the potential predictors, the power of the implanted intraocular lens (IOL) and the spherical equivalent refraction at the spectacle plane (SEQ) are known [4]. The most important benefit of empirical calculation principles is that they are independent of any specific model eye. However, to overcome the missing link between predictors a very large number of clinical cases (historic data) typically have to be evaluated [5, 6].

In contrast, theoretical-optical formulae are based on a pseudophakic model eye containing 3–5 refractive surfaces. The calculation may be performed using matrix algebra, vergence transformations, or ray tracing with restrictions to the paraxial space [1]. With theoretical-optical formulae the interaction between biometric values is used to define the formula, and subsequently some fine-tuning is required in terms of formula constant optimisation to adapt the (generally valid) formula calculation scheme to the individual characteristics of a specific IOL type [4–6]. In general, arbitrary metrics for the formula prediction error could be used for formula constant optimisation. Among these would be the mean, mean absolute, median, median absolute, root mean squared error or the standard deviation or width of any confidence interval of the error [6]. For example, the logic behind using the root mean squared error, as typically used in (linear) regression analysis or in cost functions of neural network applications, is to penalise large errors more than small errors.

Numerical raytracing cannot be formulated in terms of linear equations. Instead, a representative bundle of rays originated from a source has to be followed successively through all refractive surfaces and optical interspaces to the image plane where a sharp focus is to be formed. Numerical raytracing requires detailed information on the exact shape of all refracting surfaces, the relative positioning of the surfaces, the refractive indices of all ocular media (cornea, aqueous humor, the IOL and the vitreous humour), and the shape and location of the aperture stop. These constraints can sometimes make clinical application in a routine setup inconvenient.

All strategies based on pseudophakic model eyes (theoretical formulae as well as numerical raytracing) require an (empirical) prediction of the axial IOL position [7, 8], whereas raytracing always deals with the ‘geometric’ position of the IOL (ALP) within the eye. In contrast, theoretical-optical formulae involve either the geometrical or a fictitious axial position (the so-called ‘effective’ lens position ELP) depending on the formula architecture [7, 8].

To simplify the calculations with theoretical-optical formulae several internal constants or parameters, regressions, or hard / soft truncations of biometric measures are introduced in the IOL power formula architecture. These are mostly based on empirical work of the formula authors [4, 5]. Since in most formulae the cornea is considered as a thin lens model instead of as a meniscus lens having two refractive surfaces, we use a keratometer index to convert the biometrically measured corneal front surface radius of curvature (Ra) to a corneal power. This conversion is primarily based on assumptions about the ratio of corneal front to back surface radius as well as the central corneal thickness, and these cannot be verified by the IOL calculation formula or any keratometer [3]. Especially in patients with a history of corneal refractive surgery this conversion systematically fails.

The keratometer index in particular is well known as a relevant source of errors in IOL power calculation or prediction of the postoperative refraction, even in ‘normal’ eyes. Some formulae use an explicit keratometer index such as the Javal index (nK = 1.3375, Holladay 1 formula, [9]). Others use a ‘customised’ keratometer index (nK = 1.3315, Haigis formula [10]; nK = 1.333, SRK/T formula [11, 12]) or do not explicitly specify nK (Hoffer Q formula [13, 14])). Some newer generation formulae consider the cornea as a thick lens model (meniscus lens) having 2 refractive surfaces. In these models, the conversion to corneal power requires the radii of the corneal front and back surfaces together with the corneal thickness and the refractive index of the cornea. As quoted in the literature, this last parameter varies with values in the region of nC = 1.376 (Olsen formula [3, 8]; Castrop formula, [15, 16]). Since most of the conversion formulae systematically overestimate the corneal power (especially with the Javal keratometer index nK = 1.3375, depending on the corneal radius), the axial position has to be shifted backwards to compensate in order to obtain an appropriate IOL power [7, 8, 15]. However, this shift in axial lens position has a much larger effect with high power IOLs than with low power IOLs. With a zero lens axially shifting the IOL has no effect.

The **purpose of the present study** is

- to investigate the performance of 3 classical theoretical-optical lens power calculation formulae (Holladay 1, Hoffer Q, and Haigis formula) based on a thin lens cornea together with a modern formula (Castrop formula) based on a thick lens cornea in 2 large clinical datasets with aspherical (aberration correcting) hydrophobic acrylic intraocular lenses,
- to isolate the lens power calculation part of the IOL power formula from the lens position prediction term and to vary the keratometer index (Holladay 1, Hoffer Q, and Haigis formula) or the corneal refractive index (Castrop formula) together with the formula constants (pACD for Hoffer Q, SF for Holladay 1, a0/a1/a2 for Haigis, C/H/R for Castrop) in order to minimise the formula prediction error,
- to determine the trend error of the corneal front surface radius of curvature and to zero this trend error by modulating the above mentioned refractive indices and formula constants, and
- to cross-validate the results for the ‘optimal’ refractive index (either keratometer or corneal refractive index) by using the indices derived from one dataset and applied to the other dataset and evaluating the formula performance using these cross-validated refractive indices.

## Materials and methods

### Dataset for this study

In this retrospective study we analysed 2 datasets containing measurements from eyes from a cataract population performed at Augen- und Laserklinik Castrop-Rauxel, Castrop-Rauxel, Germany. The data were anonymised at source and transferred to us in an anonymous fashion. The local ethics committee provided a waiver for this study (Ärztekammer des Saarlandes, registration number 157/21), and patient informed consent was not required.

Dataset 1 contains data from 888 eyes (490 right eyes and 398 left eyes; 495 female and 393 male) of 888 patients with a mean age of 71.2±9.1 years (median: 71 years, range: 47 to 91 years). Each patient received a monofocal hydrophobic acrylic aberration-correcting aspheric lens (Vivinex, Hoya Surgical Optics, Singapore). Dataset 2 contains data from 822 eyes (418 right eyes and 404 left eyes; 433 female and 389 male) of 822 patients with a mean age of 72.7±9.6 years (median: 72 years, range: 49 to 94 years), each of whom received a monofocal hydrophobic acrylic spherical lens (SA60AT, Alcon, Fort Worth, USA).

The datasets contained preoperative biometric data derived with the IOLMaster 700 (Carl-Zeiss-Meditec, Jena, Germany). The measured parameters included: axial length AL, central corneal thickness CCT measured from epithelium to endothelium, anterior chamber depth ACD measured from the corneal front apex to the anterior apex of the crystalline lens, central lens thickness LT, and the corneal front surface radius measured in the flat (R1) and in the steep meridian (R2). In addition, the refractive power of the inserted lens (PIOL) and the postoperative refraction (sphere and cylinder) 5 to 12 weeks after cataract surgery were measured by an experienced optometrist and recorded in the datasets. The datasets included only data with a postoperative Snellen decimal visual acuity of 0.8 (20/25 Snellen lines) or higher to ensure that the postoperative refraction was reliable. The descriptive data on pre-cataract biometry, PIOL and postoperative refraction are summarised in **Table 1**.

**Table 1:** Descriptive statistics of the 2 datasets with mean, standard deviation (SD), median, the lower (quantile 2.5%) and upper (quantile 97.5%) boundary of the 95% confidence interval, and the interquartile range IQR (quartile 75%—quartile 25%). AL refers to the axial length, CCT to the central corneal thickness, ACD to the external phakic anterior chamber depth measured from the corneal front apex to the front apex of the crystalline lens, LT to the central thickness of the crystalline lens, R1 and R2 to the corneal radii of curvature for the flat and steep meridians, Rmean to the average of R1 and R2, PIOL to the refractive power of the intraocular lens implant, and SEQ to the spherical equivalent power achieved 5 to 12 weeks after cataract surgery.

Dataset 1: 888 eyes with HOYA Vivinex IOL									
	AL in mm	CCT in μm	ACD in mm	LT in mm	R1 in mm	R2 in mm	Rmean in mm	PIOL in dpt	SEQ in dpt
Mean	24.0980	559	3.1864	4.6176	7.8598	7.6732	7.7665	20.6222	-0.5612
SD	1.4072	36	0.4081	0.4568	0.2828	0.2745	0.26882	3.7318	0.9239
Median	23.9026	559	3.1848	4.5929	7.8473	7.6735	7.7654	21.0	-0.2500
Quantile 2.5%	22.0997	499	2.5139	3.8630	7.3955	7.2234	7.3137	13.5	-2.3800
Quantile 97.5%	26.7788	618	3.8299	5.3548	8.3355	8.1504	8.2280	26.0	0.3800
IQR	1.7474	49	0.5719	0.5978	0.3540	0.3717	0.3455	4.5	0.6300
Dataset 2: 822 eyes with Alcon SA60AT IOL									
Mean	23.1501	556	3.0459	4.6170	7.7806	7.6175	7.6991	22.7293	-0.4809
SD	1.5130	36	0.4048	0.4347	0.2719	0.2740	0.2654	4.5979	0.7197
Median	23.1850	555	3.0260	4.6097	7.8100	7.6400	7.7300	22.5	-0.2500
Quantile 2.5%	20.7920	493	2.3862	3.9680	7.2760	7.1060	7.2340	16.5	-2.1250
Quantile 97.5%	25.7140	614	3.7096	5.3500	8.2000	8.0500	8.1220	31.0	0.3750
IQR	1.5800	47	0.5620	0.5600	0.3500	0.3600	0.3450	4.0	0.7500

The anonymised Excel data (.xlsx-format) were imported into Matlab (Matlab 2021a, MathWorks, Natick, USA) for further processing.

### Preprocessing of the data

Custom software was written in Matlab. The patient age was derived from the date of cataract surgery and date of birth. The mean corneal radius of curvature Rmean was calculated as Rmean = ½·(R1+R2). We additionally calculated R = 0.5·R1·R2/(R1+R2), as some formulae (e.g. SRK/T or Hoffer Q formula) deal with the corneal radius reconverted from the mean corneal power. The spherical equivalent refraction SEQ was derived as SEQ = sphere + 0.5·cylinder. The following lens power calculation formulae were considered in this constant optimisation process:

- Hoffer Q formula published by Hoffer [13, 14, 17],
- Holladay 1 formula published by Holladay and Prager [9],
- Haigis formula [10], as well as the
- Castrop formula published by Wendelstein et al. and Langenbucher et al. [15, 16].

The Hoffer Q formula considers the AL and Kmean (keratometer index nK = 1.3375) together with the formula constant pACD. In the Q part of the formula for calculation of the ELP some truncation is performed for the AL.

The Holladay 1 formula considers the AL and the Rmean together with the formula constant SF. In the formula, there appears some truncation in R and a nonlinear transformation in AL. The keratometer index used in the Holladay formula is 4/3.

The Haigis formula considers the AL, ACD and Rmean together with a formula constant triplet a0/a1/a2. The 3 formula constants are used in terms of a multilinear regression to calculate the ELP to ELP = a0+a1·ACD+a2·AL. There are no truncations or linear/nonlinear transformations, and the keratometer index used in the Haigis formula is nK = 1.3315.

The Castrop formula considers the AL, CCT, ACD, LT, Rmean, and the corneal back surface radius together with a formula constant triplet C/H/R. For simplicity (as outlined in [5, 6, 15]) the measurement of the corneal back surface radius can be replaced in the case of ‘normal’ cataracts without any corneal pathology or a history of corneal refractive surgery by a value of 0.834·Rmean. The formula constants C and H are used in terms of a multilinear regression to calculate the ELP, and R is used as an offset in the formula predicted refraction to account e.g. for the refraction lane distance. There are no truncations in the predictors, and the axial length is linearly transformed with a sum-of-segments concept according to Cooke et al. [18, 19]. The corneal refractive index used in the Castrop formula is nC = 1.376.

All formulae included in this analysis were reorganised and solved for the SEQ as a function of preoperative biometrical data and PIOL. The formula prediction error PE was defined as the difference between the achieved SEQ (from the postoperative follow-up examination) and the SEQ predicted by the formula. A linear regression of PE was calculated for R (Hoffer Q formula) or Rmean (Holladay 1, Haigis, or Castrop formula) in terms of minimising the root mean squared error, and the regression parameters intercept and slope were documented.

### Target criteria and formula constant optimisation

In this study for both datasets four situations have been considered for our formula constant optimisation:

- A standard optimisation of the formula using the keratometer index or corneal refractive index as proposed by the formula authors. The formula constants pACD, SF, a0/a1/a2 and C/H/R were optimised for the Hoffer Q, Holladay 1, Haigis, and Castrop formula to minimise the sum of squared formula prediction error PE.
- A formula constant optimisation where the formula constants were optimised together with the keratometer index (Hoffer Q, Holladay 1 and Haigis formula) or the corneal refractive index (Castrop formula) in terms of minimising the sum of squared formula prediction error PE.
- A formula constant optimisation where the formula constants were optimised together with the keratometer index (Hoffer Q, Holladay 1 and Haigis formula) or the corneal refractive index (Castrop formula) in terms of minimising the sum of squared formula prediction error PE and the slope of the linear regression of PE over R (Hoffer Q) or Rmean (Holladay 1, Haigis, and Castrop).
- A standard optimisation according to A) but with a keratometer index (Hoffer Q, Holladay 1, Haigis) or corneal refractive index (Castrop) as derived in the optimisation of B) with the other dataset. This means that the refractive / keratometer index values derived from dataset 1 were used for cross-validation of dataset 2 and vice versa.

To summarise, the Hoffer Q, Holladay 1, and Haigis formulae each involve a thin lens model cornea and a well-documented keratometer index. In contrast, the Castrop formula is a modern formula using a thick lens model cornea where the corneal refractive index is used instead of a keratometer index. These IOL power formulae were split into a part for the prediction of the axial lens position (empirical prediction of ELP or ALP) and the main part of the formula (dealing with paraxial vergence transformation) to derive the IOL power. For all of the optimisations that consider a variation of the keratometer index or corneal refractive index (i.e. situations B and C), the variation was restricted to the main part of the lens power calculation formula. No variation was made in the ELP prediction part of the formula.All optimisations were performed using an iterative nonlinear Levenberg-Marquardt algorithm [20–22], which minimises the sum of the squared model error. This is equivalent to a minimisation in terms of root mean squared error. The algorithm was set up with the following stopping criteria: a maximum number of iterations n = 1000, a threshold of 1e-16 for improvement in the merit function, and a threshold of 1e-14 for the step size between consecutive iterations.

The target parameter of our investigation was the formula prediction error, defined as the difference between the achieved refraction and the formula predicted refraction. First, for reference, we retrieved the formula constants (pACD, SF, a0/a1/a2, and C/H/R) with the original formula as published in [5, 6] (situation A). We then modified the formula by a variation of nK or nC to get an idea of whether the (mostly empirical) nK or nC value used in the formula yields the best solution (situation B). For that purpose, only the main part of the formula was modified, and the ELP / ALP prediction model was kept untouched.

### Presentation of the data

For both datasets, we evaluated the formula prediction error PE in terms of the mean, standard deviation (SD), median, lower and upper boundary of the 95% confidence interval (2.5% quantile and 97.5% quantile) and the interquartile range (IQR). Cumulative probability density functions (CDF plots) were derived to show the performance of the PE for situations A), B), C), and D) for both datasets. Scatterplots were used to show the PE trend error for the corneal radius R or Rmean, respectively, and to prove the efficiency of our optimisation in situation C) where the elimination trend error for R / Rmean was part of the optimisation process.

## Results

In general, the Levenberg-Marquardt iterative nonlinear optimisation algorithm showed a fast and stable convergence for both datasets and all four situations A) to D). Between 12 and 192 iterations (86 to 311 function evaluations) were necessary to find the best solution for the formula constants (and nK/nC).

The formula constants and nK/nC indices are listed in **Table 2** for situations A), B), C), and D) (as defined under "preprocessing"). In **Table 3** the mean, SD, median, the lower and upper boundaries of the 95% confidence interval (2.5% quantile and 97.5% quantile) and the IQR are displayed for both datasets and for situations A), B), C), and D).

**Table 2:** Optimised formula constants for the Hoffer Q (pACD), the Holladay 1 (SF), Haigis (a0/a1/a2), and Castrop formula (C / H / R). Formula constant optimisation was performed to minimise the sum of squared prediction errors PE. A) refers to the ‘classical’ formulae with standard nK/nC values, with B) the formula constants and nK/nC in the main part of the formula were varied for optimisation, with C) the formula constants and nK/nC in the main part of the formula were varied to minimise for PE and the PE trend error over corneal radius, and with D) a standard optimisation was performed using the nK/nC value from situation B) derived from the other dataset in terms of a cross-validation.

Formula constant; nK/nC	Dataset 1: N = 888 Hoya Vivinex lenses				Dataset 2: N = 822 Alcon SA60AT lenses			
	A)	B)	C)	D)	A)	B)	C)	D)
Hoffer Q	5.7356	5.1475	5.1020	5.2450	5.4091	4.9860	5.3569	4.9088
nK	1.3375	1.3313	1.3308	1.3323	1.3375	1.3323	1.3369	1.3313
Holladay 1	1.9618	1.7020	2.4337	1.9109	1.6959	1.6536	2.1759	1.4731
nK	4/3	1.3306	1.3383	1.3328	4/3	1.3328	1.3392	1.3306
Haigis a0	-0.6853	0.2644	0.4030	0.3115	-0.7516	0.1958	0.3306	0.1502
a1	0.3417	0.3254	0.2646	0.3246	0.2872	0.2694	0.2535	0.2702
a2	0.2029	0.1475	0.1357	0.1448	0.2019	0.1456	0.1272	0.1483
nK	1.3315	1.3271	1.3236	1.3269	1.3315	1.3269	1.3228	1.3271
Castrop C	0.2746	0.2722	0.2504	0.2768	0.2792	0.2762	0.2545	0.2795
H	0.3905	0.3760	0.3616	0.3749	0.1109	0.0965	0.0820	0.1094
R	0.0728	0.0544	0.0361	0.0102	0.0882	0.0698	0.0515	0.0338
nC	1.3760	1.3849	1.3959	1.3740	1.3760	1.3740	1.3839	1.3849

**Table 3:** Formula prediction error PE (difference of the SEQ measured after cataract surgery minus the formula predicted SEQ) for the Hoffer Q (pACD), the Holladay 1 (SF), Haigis (a0/a1/a2), and Castrop formula (C / H / R). SD refers to the standard deviation, 2.5% quantile and 97.5% quantile to the lower and upper boundary of the 95% confidence interval, and IQR to the interquartile range as the difference between the 75% and the 25% quantile. Formula constant optimisation was performed to minimise the sum of squared prediction errors PE. Situation A) refers to the ‘classical’ formulae with standard nK/nC values, with situation B) the formula constants and nK/nC in the main part of the formula were varied for optimisation, with situation C) the formula constants and nK/nC in the main part of the formula were varied to minimise for PE and the PE trend error over corneal radius, and with situation D) a standard optimisation was performed using the nK/nC value from situation B) derived from the other dataset in terms of a cross-validation.

Formula prediction error PE in dpt		Dataset 1: N = 888 Hoya Vivinex lenses				Dataset 2: N = 822 Alcon SA60AT lenses			
		A)	B)	C)	D)	A)	B)	C)	D)
Hoffer Q	Mean	0.0370	-0.0008	-0.0009	0.0055	0.0415	-0.0008	0.0440	-0.0086
	SD	0.4275	0.3892	0.3894	0.3903	0.4597	0.4273	0.4524	0.4284
	Median	0.0291	0.0007	-0.0027	0.0145	0.0320	-0.0016	0.0355	-0.0157
	2.5% quantile	-0.6550	-0.6064	-0.6169	-0.6169	-0.6793	-0.6625	-0.6724	-0.6656
	97.5% quantile	0.7702	0.6116	0.6150	0.6124	0.8102	0.6801	0.7943	0.6599
	IQR	0.5231	0.4808	0.4778	0.4775	0.6082	0.5673	0.5939	0.5434
Holladay 1	Mean	0.0188	0.0021	-0.0002	0.0155	0.0048	0.0005	0.0000	-0.0179
	SD	0.4256	0.4186	0.4648	0.4231	0.4440	0.4437	0.4883	0.4495
	Median	0.0057	0.0121	-0.0198	0.0004	0.0023	-0.0043	-0.0174	-0.0291
	2.5% quantile	-0.6576	-0.66572	-0.7098	-0.6563	-0.6982	-0.6844	-0.7531	-0.7133
	97.5% quantile	0.8011	0.7131	0.8534	0.7810	0.7268	0.7101	0.8244	0.6850
	IQR	0.5371	0.5106	0.5550	0.5333	0.5619	0.5635	0.5831	0.5629
Haigis	Mean	0.0334	-0.0011	-0.0008	-0.0147	0.0447	-0.0011	0.0001	-0.0016
	SD	0.4027	0.3672	0.3876	0.3728	0.4568	0.4162	0.4174	0.4162
	Median	0.0341	-0.0047	-0.0146	-0.0182	0.0440	-0.0075	-0.0070	-0.0070
	2.5% quantile	-0.6472	-0.5999	-0.6595	-0.5967	-0.7117	-0.6573	-0.6658	-0.6570
	97.5% quantile	0.7140	0.6125	0.6679	0.6099	0.7725	0.6829	0.6954	0.6831
	IQR	0.5132	0.4613	0.4762	0.4492	0.5941	0.5630	0.5689	0.5625
Castrop	Mean	0.0000	-0.0020	0.0000	-0.0001	0.0000	-0.0032	0.0000	0.0000
	SD	0.3437	0.3434	0.3414	0.3433	0.4065	0.4061	0.4018	0.4061
	Median	-0.0085	-0.0125	-0.0113	-0.0075	-0.0130	-0.0163	-0.0101	-0.0125
	2.5% quantile	-0.5610	-0.5593	-0.5453	-0.5622	-0.6855	-0.6784	-0.6677	0.6861
	97.5% quantile	0.5581	0.5505	0.5443	0.5553	0.6747	0.6734	0.6818	0.6722
	IQR	0.4376	0.4382	0.4133	0.4363	0.5518	0.5532	0.5364	0.5516

**Fig 1:**
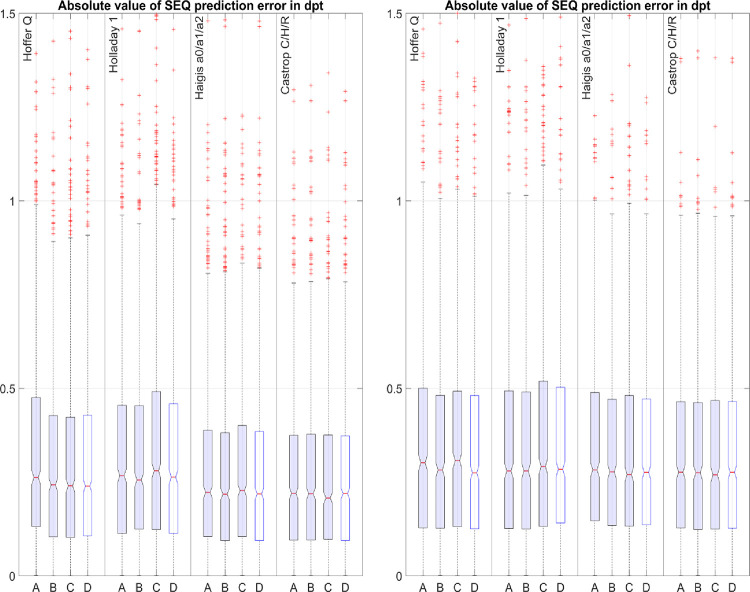
Boxplots of the absolute value of prediction error (PE, measured spherical equivalent refraction–formula predicted refraction) for the Hoffer Q, Holladay1, Haigis, and Castrop formulae for dataset 1 (Hoya Vivinex, N = 888, left graph) and dataset 2 (Alcon SA60AT, N = 822, right graph). Formula constant optimisation was performed to minimise the sum of squared prediction errors PE: Situation A refers to the ‘classical’ formulae with standard nK/nC values; with situation B the formula constants and nK/nC in the main part of the formula were varied for optimisation; with situation C the formula constants and nK/nC in the main part of the formula were varied to minimise for PE and the PE trend error over corneal radius; with situation D) a standard optimisation was performed using the nK/nC value from situation B derived from the other dataset in terms of a cross-validation.

**Fig 1** displays the boxplot of the absolute value of the PE for both datasets (left graph: dataset 1, Hoya Vivinex lens, N = 888; right graph: dataset 2, Alcon SA60AT lens, N = 822) and situations A), B), C), and D). The boxes refer to the 25% to 75% quartile, the red lines in the boxes to the median value, and the whiskers to the lower and upper boundaries of the 95% confidence interval (threshold of the outliers). Under variation of nK/nC the performance of the Hoffer Q formula was increased in both datasets (comparing 1^st^ (A) and 2^nd^ box (B) in each section). With the Haigis formula there was only a very slight improvement and with the Holladay 1 and the Castrop formula there was no systematic improvement. Comparing the 3^rd^ box (C) in each section to the 2^nd^ box (B) we can see that with the Hoffer Q formula and with the Castrop formula, that including the trend error for corneal radius in the optimisation strategy does not degrade the overall performance. In contrast, especially with the Holladay formula and very slightly with the Haigis formula, including the corneal radius trend error of PE in the optimisation strategy results in a deterioration of the absolute prediction error. Using the nK/nC value derived from dataset 2 for optimising the formula constants for dataset 1 (situation D, 4^th^ box in each section, left graph) or using the nK/nC value derived from dataset 1 for optimising the formula constants for dataset 2 (situation D, 4^th^ box in each section, right graph) does not systematically affect the outcome of the formula compared to the ‘optimal’ value (derived in situation B, 2^nd^ box in each section, left and right graph).

**Fig 2:**
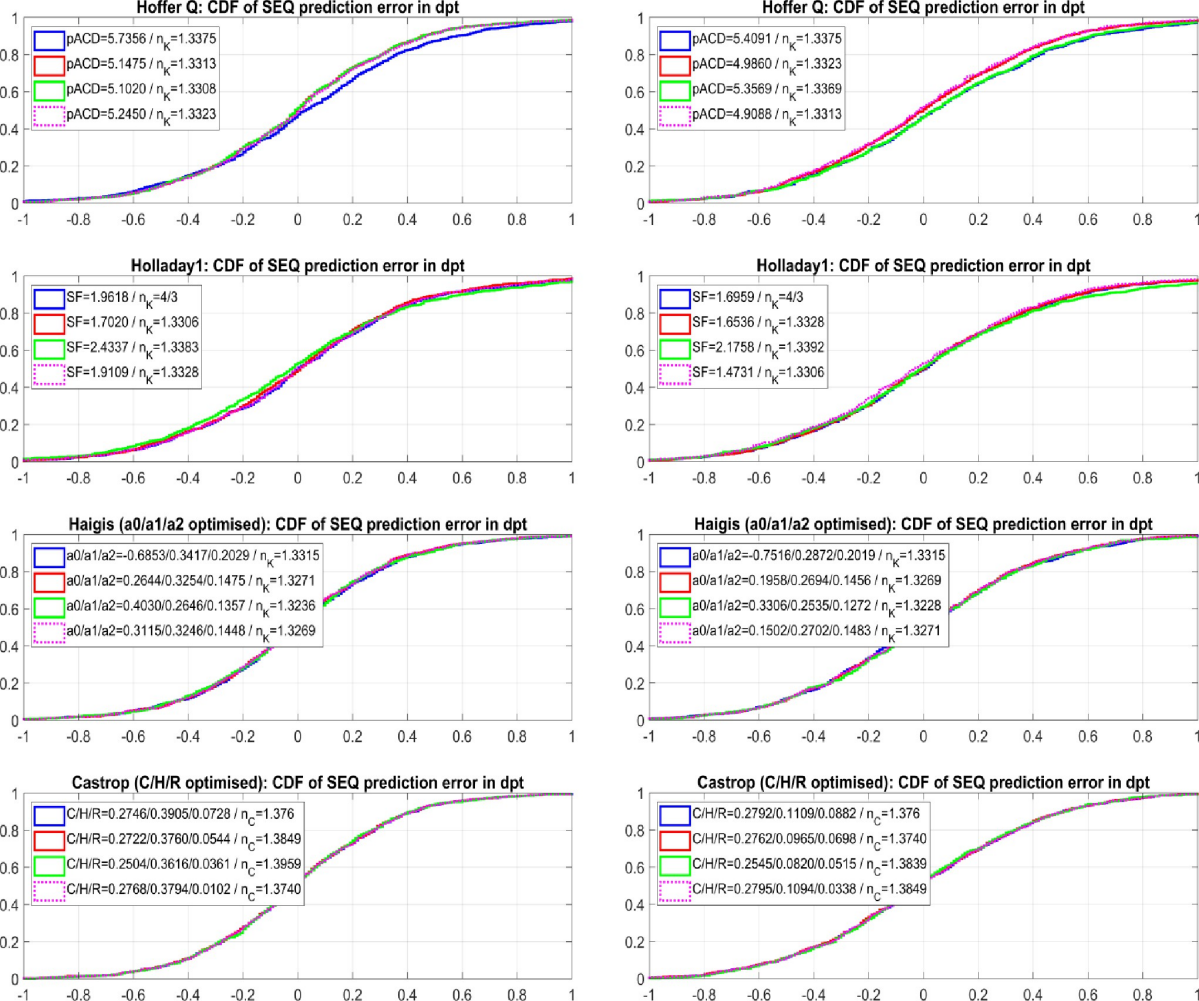
Cumulative density function (CDF on the Y axis) of prediction error (PE on the X axis, measured spherical equivalent refraction–formula predicted refraction) for the Hoffer Q, Holladay1, Haigis, and Castrop formula for dataset 1 (Hoya Vivinex, N = 888, left graph) and dataset 2 (Alcon SA60AT, N = 822, right graph). The ticks on the Y axis indicate the portion of cases in the dataset showing a PE less equal this mark. The steeper the slope around a PE = 0 the more cases within the respective limits. The number of eyes within PE limits (PE within ±0.5 dpt) could be extracted from the graph by subtracting the respective CDF values at PE = 0.5 dpt and PE = -0.5 dpt. Formula constant optimisation was performed to minimise the sum of squared prediction errors PE: Situation A) (blue lines) refers to the ‘classical’ formulae with standard nK/nC values; with situation B) (red lines) the formula constants and nK/nC in the main part of the formula were varied for optimisation; with situation C) (green lines) the formula constants and nK/nC in the main part of the formula were varied to minimise for PE and the PE trend error over corneal radius; with situation D) (dashed magenta lines) a standard optimisation was performed using the nK/nC value from situation B) derived from the other dataset in terms of a cross-validation.

**Fig 2** shows the cumulative density function (CDF) plot of PE for both datasets for the Hoffer Q, Holladay1, Haigis, and Castrop formulae for dataset 1 (Hoya Vivinex, N = 888, left graph) and dataset 2 (Alcon SA60AT, N = 822, right graph). We can see from the graphs that the performance of the Castrop formula does not differ for situations A), B), C), and D) for either dataset. The Haigis formula shows very slight differences in the performance curve comparing situations A) to D).On the other hand, the Hoffer Q formula especially, and also the Holladay formula to a certain extent, both show systematic differences between the 4 situations A) to D). The steeper slopes of the red lines (situation B) compared to the blue lines (situation A) indicate that the performance of the formulae could in general be improved by variation of the keratometer index, particularly with the Hoffer Q formula.

**Fig 3:**
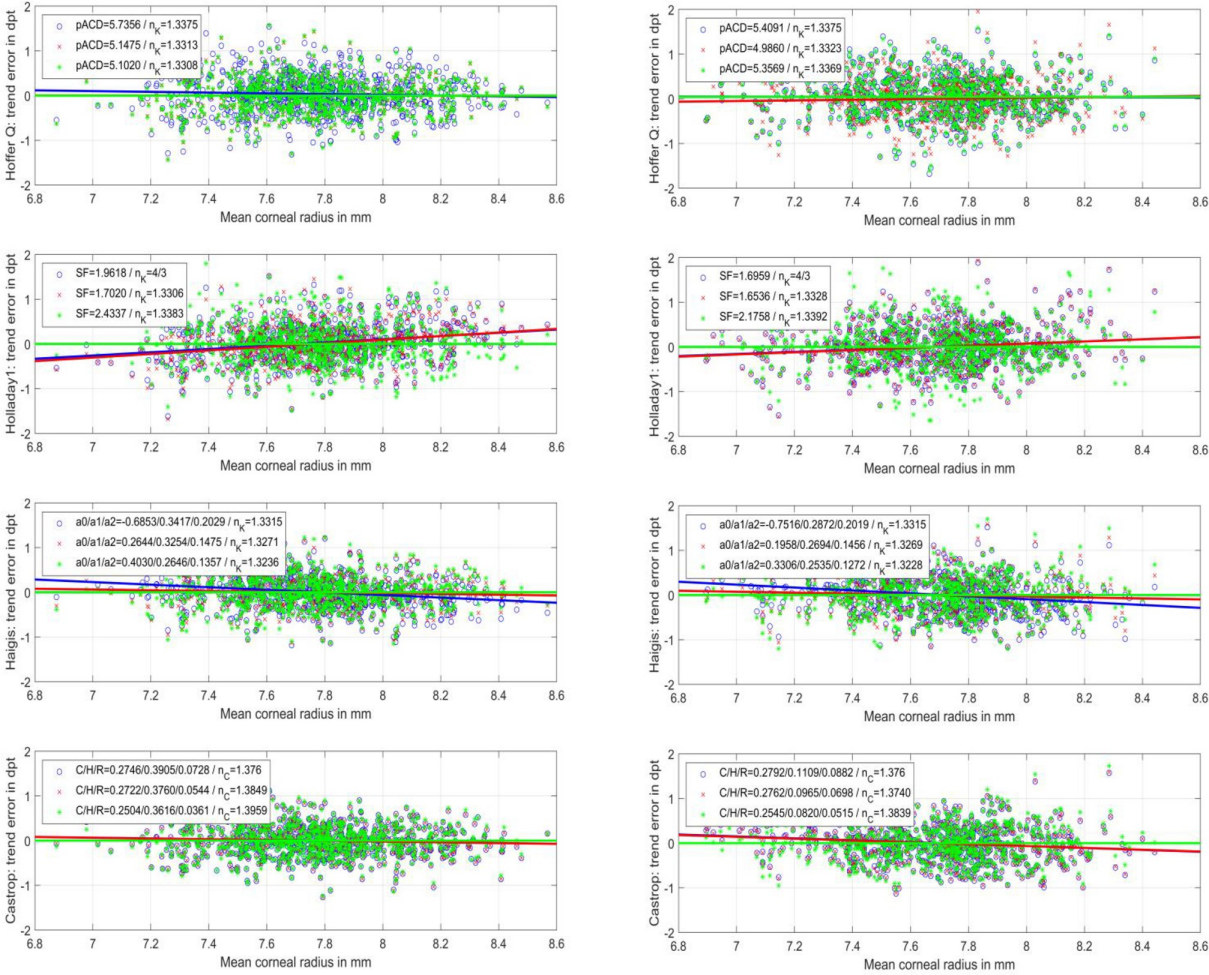
Scatterplot of prediction error (PE, measured spherical equivalent refraction–formula predicted refraction) versus the corneal radius for the Hoffer Q, Holladay1, Haigis, and Castrop formula for dataset 1 (Hoya Vivinex, N = 888, left graph) and dataset 2 (Alcon SA60AT, N = 822, right graph). Formula constant optimisation was performed to minimise the sum of squared prediction errors PE: Situation A) (blue o-markers and blue line indicating the trend error derived from linear regression) refers to the ‘classical’ formulae with standard nK/nC values; with situation B) (red x-markers and red line indicating the trend error derived from linear regression) the formula constants and nK/nC in the main part of the formula were varied for optimisation; with situation C) (green *-markers and green line indicating the trend error derived from linear regression) the formula constants and nK/nC in the main part of the formula were varied to minimise for PE and the PE trend error over corneal radius. Situation D) is not shown in this figure.

**Fig 3** displays the scatterplot of PE for both datasets for the Hoffer Q, Holladay1, Haigis, and Castrop formulae for dataset 1 (Hoya Vivinex, N = 888, left graph) and dataset 2 (Alcon SA60AT, N = 822, right graph). The slopes of the regression lines in the plots represent the trend error of PE for the corneal radius R/Rmean. We can see from the graphs (blue regression lines) that the Hoffer Q formula (slope: -0.0837/-0.0090 dpt/mm for dataset 1/2) has a negligible trend error for corneal radius, whereas the Holladay 1 formula (slope: 0.3630/0.2343 dpt/mm for dataset 1/2) shows a positive trend error. The Haigis formula (slope: -0.2889/-0.3249 dpt/mm for dataset 1/2) and the Castrop formula (slope: -0.0852/-0.2147 dpt/mm for dataset 1/2) both show a slight negative trend error. Including nK/nC in the optimisation process did not worsen the trend error of PE for corneal radius (red regression lines) for any of the formulae under test. We see from the graphs that the Hoffer Q formula (slope: -0.0056/0.0713 dpt/mm for dataset 1/2) again has a negligible trend error for corneal radius. Conversely the Holladay 1 formula (slope: 0.4003/0.2426 dpt/mm for dataset 1/2) shows a positive trend error and the Haigis formula (slope: -0.0828/-0.1077 dpt/mm for dataset 1/2) and the Castrop formula (slope: -0.0851/-0.2057 for dataset 1/2) a slight negative trend error. For all formulae under test, optimising for the sum of squared PE and the trend error of PE for corneal radius R/Rmean was able to nullify the trend error (situation C, green regression lines). However, especially with the Holladay 1 formula (and slightly for the Haigis formula), elimination of the trend error for R/Rmean is at the cost of the overall performance (compare **Fig 1** situation (C) vs. situation (B).

## Discussion

Numerous formulae for calculating intraocular lens power have been proposed in the last 20 years. In contrast to the basic formulae of Fyodorov [23] or Gernet [24] or the classical formulae of Sanders, Retzlaff and Kraff (SRK/T), Hoffer (Hoffer Q), Holladay (Holladay 1) or Haigis, most of the formula authors nowadays do not disclose or publish the calculation strategy. At best they offer WEB based applications or software solutions for calculating the lenses. Such software tools do not allow batch calculations on a large set of patient data. Today, classical formulae are increasingly being replaced by ‘modern’ calculation strategies such as the Barrett Universal II, Kane, Pearl, EVO, VRF/VRF-G, Hill RBF, K6, or T2 formulae in many countries of the world [1, 2]. To compare the prediction performance with other formulae it is necessary to enter the data from preoperative data (biometry), intraoperative data (lens power) and postoperative data (manual refraction after 4 weeks to 6 months) manually, introducing a large risk of transcription errors. Additionally, a systematic optimisation of constants is not possible for undisclosed formulae [1, 3].

In all lens power calculation formulae, the corneal power has to be known but it cannot be measured directly. Using manual keratometers or automated keratometers or topographers integrated in the biometer device we normally measure the corneal front surface radius. With some modern OCT based biometers it is also possible to assess the central corneal thickness and the corneal back surface radius, and this enables us to switch to a thick lens model for the cornea. However, in most lens power calculation formulae, even modern ones, the cornea is still considered as a thin lens, and this requires a keratometer index for conversion of corneal radius to power. The CCT and corneal back surface curvature are ignored [15]. All keratometer indices are implicitly based on a corneal model (e.g. based on a corneal refractive index, the CCT and the ratio of corneal front to back surface radius) and do not represent a proper conversion from corneal radius to power in the general case. For example, if we consider the Javal keratometer index nK = 1.3375 and a corneal front / back surface radius of 7.7 / 6.8 mm and a corneal thickness of 500 μm (refractive index of aqueous humour 1.336) according to the Gullstrand schematic model eye, the corneal refractive index nC should be nC = 1.3229 / 1.3760 / 1.3210 if the keratometric corneal power 337.5/7.7 dpt = 43.8312 dpt represents the equivalent power / back vertex power / front vertex power. If we compare these refractive indices to the corneal refractive index listed in schematic model eyes, it is clear that the Javal keratometer index for ‘normal corneas’ represents the back vertex power, which has no impact in IOL power calculation. Instead, as all distances in the eye are referenced to the corneal front apex (e.g. AL, CCT, ACD) the corneal front vertex power has to be used in theoretical-optical formulae considering the cornea as thin lens and therefore the use of e.g. the Javal keratometer index is questionable.

Bearing these data in mind, the basic idea behind the present study was to investigate the impact of the keratometer index or corneal refractive index used in classical IOL power calculation formulae and in a modern fully disclosed formula. The optimisations for each formula and each situation were carried out as described in "Target criteria and formula constant optimisation". As we can see from **Fig 1**, the Hoffer Q formula mostly benefits from a variation of nK, whereas in the other formulae there was no improvement or only a negligible improvement with variation of nK/nC. The respective metrics are listed in **Table 3**. **Fig 1** also shows that, especially with dataset 1 (the aspheric aberration correcting Hoya Vivinex lens), the Haigis and Castrop formulae perform much better compared to the Hoffer Q or Holladay 1 formulae with or without variation of nK/nC. In contrast, in dataset2 (spherical lens) there is no clinically relevant difference between the four formulae. This difference between the two datasets (from the same clinical centre) could be a result of the (on average) larger spherical aberrations after implantation of the spherical Alcon lens in dataset 2 as compared to the aberration-correcting Hoya lens in dataset 1. This could affect the precision of postoperative refractometry and may hide the differences between formulae in dataset 2. From **Fig 3** we can also see that the trend error of PE for the corneal radius (situation C) could be eliminated completely with variation of the formula constants and nK/nC. However, **Fig 1** shows us that eliminating the trend error significantly/slightly worsens the performance of the Holladay 1 / Haigis formula (comparing situations B and C), whereas the Hoffer Q and the Castrop formula are mainly unaffected. This is obvious as the latter formulae initially show a low trend error. Overall, even with the Hoffer Q formula, which uses the Javal keratometer index for calculation (where we would expect a large trend error), in reality this trend error in PE for corneal radius is surprisingly low. This might be mostly due to the concept for prediction of the axial lens position ELP, which might compensate for the trend error in R. This ELP prediction model was not modified in our analysis [25]. However, we should be aware that modifying the formulae by variation of the nK/nC and/or including the trend error of PE for the corneal radius in the optimisation strategy requires new formula constants, as shown in **Table 2**.

In the past, several studies addressing the trend error of formulae with respect to the axial length have been published [25]. In the WEB we find some recommendations as to which formula is most suitable for long or short eyes. However, in practice, making recommendations for ‘suitable’ or ‘unsuitable’ formulae based only on the axial length is only half of the truth: the combinations of all biometric measures have to be considered in such recommendations. Especially the ‘uncommon’ combinations of long eyes with steep corneas or short eyes with flat corneas seem to be particularly challenging.

Therefore, in the present study we tried to find out whether classical formulae (e.g. the Hoffer Q, Holladay 1, or Haigis formula) could be ‘tuned’ for modern IOLs and cataract surgery techniques by means of a variation of the keratometer index, or whether modern formulae with a thick lens model for the cornea (e.g. the Castrop formula) could be improved by a variation of the corneal refractive index. This seems to be true for some of the formulae (principally the Hoffer Q formula). Even if we apply the nK/nC value derived from dataset 2 for dataset 1 or vice versa (in terms of cross-validation), the benefit might be visible if compared to the performance using the original nK/nC (comparing situation B to A or situation D to A in **Fig 1**). However, eliminating the trend error of PE for the corneal radius with variation of nK/nC and the formula constants, the performance of the Holladay 1 formula which showed the largest (positive) trend and the Haigis formula (which showed some negative trend) deteriorated somewhat (comparing situation C to situation A or B). This might be mostly due to the fact that we restricted the modifications made in the formula (variation of nK/nC) to the main part of the formula (the vergence transformation) while keeping the empirical part (prediction of the ELP/ALP) unchanged. As the empirical ELP/ALP prediction should be balanced in all the IOL power calculation formulae to achieve the best results, the ELP/ALP prediction strategy might be included in the modification, especially in formulae where the corneal radius is a predictor for the axial lens position.

However, there are some limitations in our study: firstly, we considered only 2 datasets with hydrophobic acrylic lenses, one with an aberration correcting aspheric and one with a spherical lens design. Even though these are very popular lenses on the market, they might not reflect the full spectrum of lens designs. Secondly, we optimised all the formula constants (and nK/nC) for the sum of squared formula prediction error PE, which is equivalent to a minimisation of the root mean squared PE or the ‘energy’ of the PE [5, 6]. Even if we feel that this metric is the most suitable target criterion, there might be a variety of other target parameters or metrics for optimisation. Thirdly, in our study we modified only the main part of the IOL power calculation formulae keeping the ELP/ALP prediction concept unchanged. The Hoffer Q and the Holladay 1 formulae consider the corneal curvature in the ELP prediction concept, whereas the Haigis and the Castrop formula do not. The results might change slightly if the variation in nK/nC were also considered in the ELP prediction part of the formulae. And last but not least, the potential trend error for the axial length was not considered for our analysis.

## Conclusion

This study describes techniques for modification of three classical IOL power calculation formulae (Hoffer Q, Holladay 1, Haigis) and a fully disclosed modern formula (Castrop formula) by variation of the keratometer index nK (with a thin lens model for the cornea) or the corneal refractive index nC (with a thick lens model of the cornea). Some of the formulae benefit from variation of nK/nC, whereas others do not. However, we have to be aware that variation of nK/nC always requires modification of the formula constants. The study was based on 2 large datasets with hydrophobic acrylic lenses, one with an aberration correcting aspheric design (Hoya Vivinex) and one with a spherical design (Alcon SA60AT). More clinical data are required to verify our findings and to derive recommendations on modifications of the nK/nC.

## References

[1] Melles RB, Kane JX, Olsen T, Chang WJ. Update on intraocular lens calculation formulae. Ophthalmology 2019; 126(9):1334–1335. doi: 10.1016/j.ophtha.2019.04.011 30980854

[2] Savini G, Taroni L, Hoffer KJ. Recent developments in intraocular lens power calculation methods-update 2020. Ann Transl Med. 2020;8(22):1553. doi: 10.21037/atm-20-2290 33313298 PMC7729321

[3] Shammas HJ. Intraocular lens power calculations. Slack Inc 2004. ISBN-13: 978–1556426520.

[4] Aristodemou P, Knox Cartwright NE, Sparrow JM, Johnston RL. Intraocular lens formula constant optimization and partial coherence interferometry biometry: Refractive outcomes in 8108 eyes after cataract surgery. J Cataract Refract Surg 2011; 37(1):50–62. doi: 10.1016/j.jcrs.2010.07.037 21183099

[5] Langenbucher A, Szentmáry N, Cayless A, Müller M, Eppig T, Schröder S, et al. IOL formula constants: strategies for optimization and defining standards for presenting data. Ophthalmic Res. 2021a;64(6):1055–1067. doi: 10.1159/000514916 33530082 PMC8743903

[6] Langenbucher A, Szentmáry N, Cayless A, Wendelstein J, Hoffmann P. Strategies for formula constant optimisation for intraocular lens power calculation. PLoS One. 2022 May 5;17(5):e0267352. doi: 10.1371/journal.pone.0267352 35511906 PMC9071153

[7] Olsen T, Hoffmann P. C constant: new concept for ray tracing-assisted intraocular lens power calculation. J Cataract Refract Surg 2014; 40(5):764–773. doi: 10.1016/j.jcrs.2013.10.037 24767910

[8] Olsen T. J Prediction of the effective postoperative (intraocular lens) anterior chamber depth. J Cataract Refract Surg 2006; 32(3):419–424. doi: 10.1016/j.jcrs.2005.12.139 16631049

[9] Holladay JT, Prager TC, Chandler TY, Musgrove KH, Lewis JW, Ruiz RS. A three-part system for refining intraocular lens power calculations. J Cataract Refract Surg 1988; 14(1):17–24. doi: 10.1016/s0886-3350(88)80059-2 3339543

[10] Haigis W, Lege B, Miller N, Schneider B. Comparison of immersion ultrasound biometry and partial coherence interferometry for intraocular lens calculation according to Haigis. Graefes Arch Clin Exp Ophthalmol. 2000 Sep;238(9):765–73. doi: 10.1007/s004170000188 11045345

[11] Retzlaff JA, Sanders DR, Kraff MC. Development of the SRK/T intraocular lens implant power calculation formula. J Cataract Refract Surg 1990; 16(3):333–340. doi: 10.1016/s0886-3350(13)80705-5 2355321

[12] Sanders DR, Retzlaff JA, Kraff MC, Gimbel HV, Raanan MG. Comparison of the SRK/T formula and other theoretical and regression formulas. J Cataract Refract Surg 1990; 16(3):341–346. doi: 10.1016/s0886-3350(13)80706-7 2355322

[13] Hoffer KJ. Steps for IOL power calculation. Am Intraocul Implant Soc 1980; 6(4):370. . 7440385

[14] Hoffer KJ. The Hoffer Q formula: a comparison of theoretic and regression formulas. J Cataract Refract Surg 1993; 19(6):700–712. doi: 10.1016/s0886-3350(13)80338-0 8271165

[15] Langenbucher A, Szentmáry N, Cayless A, Weisensee J, Fabian E, Wendelstein J, et al. Considerations on the Castrop formula for calculation of intraocular lens power. PLoS One. 2021b Jun 2;16(6):e0252102. doi: 10.1371/journal.pone.0252102 34077432 PMC8172026

[16] Wendelstein J, Hoffmann P, Hirnschall N, et al. Project hyperopic power prediction: accuracy of 13 different concepts for intraocular lens calculation in short eyes. Br J Ophthalmol 2021 27: bjophthalmol-2020-318272. doi: 10.1136/bjophthalmol-2020-318272 33504489

[17] Hoffer KJ. Intraocular lens calculation: the problem of the short eye. Ophthalmic Surg 1981; 12(4):269–272. . 7254770

[18] Cooke DL, Cooke TL. A comparison of two methods to calculate axial length. J Cataract Refract Surg 2019a 45(3):284–292. doi: 10.1016/j.jcrs.2018.10.039 30851805

[19] Cooke DL, Cooke TL. Approximating sum-of-segments axial length from a traditional optical low-coherence reflectometry measurement. J Cataract Refract Surg 2019b; 45(3):351–354. doi: 10.1016/j.jcrs.2018.12.026 30851808

[20] Kanzow C, Yamashita N, Fukushima M. Levenberg–Marquardt methods with strong local convergence properties for solving nonlinear equations with convex constraints. J Comp Applied Mathematics 2004. 172 (2): 375–397. doi: 10.1016/j.cam.2004.02.013

[21] Levenberg K. A method for the solution of certain problems in least squares. Quart Appl Math 1944; 2:164–168.

[22] Marquardt D. An algorithm for least-squares estimation of nonlinear parameters. SIAM J Appl Math 1963; 11:431–441.

[23] Fyodorov SN, Galin MA, Linksz A. Calculation of the optical power of intraocular lenses. Invest Ophthalmol 1975; 14: 625–628. 1150402

[24] Gernet H, Ostholt H, Werner H. Die präoperative Berechnung intraocularer Binkhorst-Linsen. 122. Vers. d. Ver. Rhein.-Westfäl. Augenärzte. Balve, Verlag Zimmermann 1970: pp. 54–55.

[25] Zhang JQ, Zou XY, Zheng DY, Chen WR, Sun A, Luo LX. Effect of lens constants optimization on the accuracy of intraocular lens power calculation formulas for highly myopic eyes. Int J Ophthalmol 2019; 12(6):943–948. doi: 10.18240/ijo.2019.06.10 31236350 PMC6580219

